# Epidemiology of critically ill patients in intensive care units: a population-based observational study

**DOI:** 10.1186/cc13026

**Published:** 2013-09-30

**Authors:** Allan Garland, Kendiss Olafson, Clare D Ramsey, Marina Yogendran, Randall Fransoo

**Affiliations:** 1 Department of Internal Medicine, University of Manitoba, 820 Sherbrook Street, Winnipeg, Manitoba R3A 1R9, Canada; 2 Department of Community Health Sciences, University of Manitoba, 810 Sherbrook Street, Winnipeg, Manitoba R3A 1R8, Canada; 3 Manitoba Center for Health Policy, University of Manitoba Winnipeg, 408-727 McDermot Avenue, Winnipeg, Manitoba R3E 3P5, Canada

## Abstract

**Introduction:**

Epidemiologic assessment of critically ill people in Intensive Care Units (ICUs) is needed to ensure the health care system can meet current and future needs. However, few such studies have been published.

**Methods:**

Population-based analysis of all adult ICU care in the Canadian province of Manitoba, 1999 to 2007, using administrative data. We calculated age-adjusted rates and trends of ICU care, overall and subdivided by age, sex and income.

**Results:**

In 2007, Manitoba had a population of 1.2 million, 118 ICU beds in 21 ICUs, for 9.8 beds per 100,000 population. Approximately 0.72% of men and 0.47% of women were admitted to ICUs yearly. The age-adjusted, male:female rate ratio was 1.75 (95% CI 1.64 to 1.88). Mean age was 64.5 ± 16.4 years. Rates rose rapidly after age 40, peaked at age 75 to 80, and declined for the oldest age groups. Rates were higher among residents of lower income areas, for example declining from 7.9 to 4.4 per 100,000 population from the poorest to the wealthiest income quintiles (*p* <0.0001). Rates of ICU admission slowly declined over time, while cumulative yearly ICU bed-days slowly rose; changes were age-dependent, with faster declines in admission rates with older age. There was a high rate of recidivism; 16% of ICU patients had received ICU care previously.

**Conclusions:**

These temporal trends in ICU admission rates and cumulative bed-days used have significant implications for health system planning. The differences by age, sex and socioeconomic status, and the high rate of recidivism require further research to clarify their causes, and to devise strategies for reducing critical illness in high-risk groups.

## Introduction

The care of critically ill people in Intensive Care Units (ICUs) is a large [1-5], and expensive [1,3,6-11] component of modern health care. In Canada, 11% of hospitalizations include time in such units [12], and 19% of people die in them [13]. In the United States, up to half of all people experience ICU care during their final year of life [14,15], many die there [16,17], and demand is rising [11,15].

While all developed countries have extensive ICU infrastructures, there is large variability in the supply of ICU beds between and within countries [1,14]. Although the reasons for such large variations are unclear, population-based assessment of ICU utilization is a starting point towards insight into the epidemiology of critical illness, and that insight is necessary for ensuring the health care systems are able to meet current and future needs for ICU care. However, few such studies have been published. In the Canadian province of Manitoba we possess detailed information about all residents, hospitalizations, and ICU care. We used these data to analyze ICU care among all adults.

## Methods

This study was performed as part of a larger project on critical illness performed at the Manitoba Centre for Health Policy [18]. We assessed all adult residents of Manitoba admitted to provincial ICUs over the nine fiscal years 1999 to 2007 (April 1, 1999 to March 31, 2008). During this interval there were no major changes to ICU organization or admission policies. Manitoba had a 2007 population of 1.19 million, of which 56% resided in the capital, Winnipeg [19]. The only other urban area is Brandon, population 50,000. Population-based analysis was facilitated by Manitoba’s location; within 250 km of its borders there are no Canadian population centers exceeding 15,000 people, nor any Canadian medical centers with certified ICU physicians (intensivists). Thus, virtually every Manitoban who required ICU care received it in Manitoba, where they are covered by a single-payer, governmental health insurance system.

Manitoba has ICUs in urban areas and rural ICUs, however, only the former have intensivists, nurses with specialized ICU education, and the capability of caring indefinitely for patients requiring invasive mechanical ventilation or other artificial life support. We refer to these as high-intensity ICUs and this designation is synonymous with the ICUs in the Winnipeg and Brandon urban centers. In contrast, the low-intensity rural ICUs have no intensivists, can manage mechanically ventilated patients for limited intervals, and commonly transfer their sicker patients to high-intensity ICUs.

The data for this analysis came from the Manitoba Integrated Critical Care Database (MICCDB) which, as previously described, accurately identifies the existence and timing of ICU admission [18,20,21]. It links patient demographic and clinical information with administrative hospital abstracts for all provincial residents.

Correctly quantifying ICU care requires accounting for inter-ICU and/or inter-hospital transfers during such care. Transfers generate multiple MICCDB records, which must be identified and merged to construct full episodes of ICU care, and ICU-containing hospital care. This was done as previously described [21] and these episodes are the units of measure in this study. Length of stay (LOS) for episodes of care was calculated as the interval between the start of the first record and the end of the final record.

We evaluated all ICU care for Manitoba residents ≥17 years old, with final hospital discharge during the study period. Manitobans were identified by the existence of a provincial Personal Health Identification Number. In analyzing ICU patient characteristics during a given year, a person with multiple ICU episodes in that year was counted only once.

We calculated population-based rates of ICU care for each year as the number of individuals who had one or more ICU episodes during that year, divided by the number of Manitobans ≥17 years old in that year, obtained from the Manitoba Health Insurance Registry. Except for age-specific rates, we used direct age adjustment [22] (relative to the Manitoba population in 2007) unless otherwise indicated. In addition to rates for admission to all Manitoba ICUs, we also calculated rates limited to the urban, high-intensity ICUs.

We evaluated patients’ age, sex, and socioeconomic status (SES). SES was divided into 11 categories; 10 indicate the quintile (separately for urban and rural areas) of average household income by postal codes, based on the 2001 Canadian census. The eleventh category comprised those living in postal codes for which average household income is not available, predominantly nursing homes and other chronic care facilities, but also other institutions such as prisons.

Group comparisons used *t* tests or χ^2^ tests as appropriate. We assessed for differences in annual parameters over the nine years by χ^2^ tests, and assessed for trends over time using ordinary least squares regression of the nine yearly values. Temporal trends in mean or median values of individual-level parameters were assessed, respectively, using ordinary least squares or median regression of the individual values by year. Age-adjusted comparisons between sexes or SES categories were done by grouped Poisson regression with inclusion of categorized age.

This work was approved by the Health Research Ethics Board of the University of Manitoba, and the Manitoba Health Information Privacy Committee (number 2008/2009-15). It was supported by the Manitoba Department of Health. All analyses were conducted using the data repository housed at the Manitoba Centre for Health Policy. Statistical analysis was done using SAS 9.2 (SAS Institute, Inc., Cary, NC, USA). Values are presented as mean ± SD unless otherwise stated. *P* values less than 0.05 were considered significant.

## Results

### ICU bed supply and utilization

In 2007, Manitoba had 118 ICU beds in 21 ICUs, contained in 16 hospitals. Ninety-one ICU beds (77%) were located in the 12 high-intensity ICUs in the seven urban, acute care hospitals. Five of the high-intensity ICUs were unspecialized units of six to nine beds, caring for medical, surgical and cardiac patients. Of the remaining seven high-intensity ICUs, one each were medical, surgical/trauma, medical-surgical and cardiac surgical (each of 10 beds), two were dedicated coronary care units (three and six beds each), and one was a six-bed respiratory ICU. Nine rural hospitals contained the other 27 ICU beds (23%), each with a single, unspecialized ICU of two to four beds. Using population data from 2007 [19], there were 9.8 ICU beds per 100,000 population for the whole province, and 13.4 in the Winnipeg metropolitan area.

During the nine-year study period, 41,833 unique Manitobans experienced 54,140 distinct episodes of ICU care, contained within 51,255 hospital episodes (ICU-containing hospital episodes). Of these individuals, 6,601 (15.8%) received ICU care during more than one hospital episode within the nine-year study period. Five percent of ICU-containing hospital episodes contained more than one distinct episode of ICU care. Inter-ICU transfers occurred within 5,205 ICU episodes (9.6%). Of the 54,140 ICU episodes, 10,060 (18.6%) took place entirely in low-intensity rural ICUs, while 1,010 (1.9%) patients started out in rural ICUs and were transferred to a high-intensity urban ICU.

Cumulative ICU use changed over the study period (Table 1). Relative to the average of 6,016 ICU episodes/year, the annual number declined by 1.5%/year (*P* = 0.02), though the entirety of this change occurred from 1999 to 2001. Cumulative ICU bed-days/year averaged 23,628 over the study period, gradually rising 1.3%/year (*P* = 0.01). These changes compare with an average 0.6%/year increase in the provincial population [19]. The mean LOS for individual ICU episodes was 3.93 days (standard deviation (SD) 6.97, median 2.1, interquartile range (IQR) 1.0 to 4.1 days) over the study period, and it rose 2.8% (0.11 days) per year (*P* <0.0001). Mean and median LOS for ICU episodes restricted to the low-intensity ICUs were significantly shorter than for those including care in high-intensity ICUs (mean values 2.22 ± 2.45 vs. 4.32 ± 7.58 days, *P* <0.0001; median values 1.7 vs. 2.4, *P* <0.001).

**Table 1:** ICU utilization, by year

***Year***	***No. of ICU episodes***	***Cumulative ICU bed-days***
1999	6,340	22,023
2000	6,677	23,889
2001	6,231	23,155
2002	5,789	22,632
2003	5,907	22,775
2004	5,758	24,365
2005	5,759	24,012
2006	5,787	25,139
2007	5,892	24,664
Yearly average	6,016	23,628

The rural, low-intensity ICUs accounted for 10.3% of total ICU bed-days over the entire study period. But even as annual provincial ICU bed-days increased, rural ICU bed-days declined an average of 4.5% per year (*P* <0.001).

Putting ICU care within the larger context of hospital bed use, 7.7% of all hospital episodes for adult Manitobans contained ICU care. ICU bed-days comprised 2.3% of all hospital bed-days over the study period, with no trend over time (*P* = 0.32). Among ICU-containing hospitalizations, the mean fraction of bed-days spent in ICUs was 31.3%, which also did not change over time (*P* = 0.55).

### Patient sex and age

With individuals as the unit of measure, a majority of ICU patients in each year were males, ranging from 58.5 to 60.8% over the nine years, without significant differences over time (*P* = 0.52). The overall mean age was 64.5 ± 16.4 years, and it decreased slightly over the study period, by 0.22 years of age/year (*P* <0.0001, in Additional file 1: Table S1). Overall, there was no difference in the mean age of patients admitted to the urban, high-intensity ICUs compared to those admitted to rural, low-intensity ICUs (64.5 ± 16.0 vs. 64.6 ± 18.1, *P* = 0.61). The women admitted to ICUs were slightly older than the men (mean 66.1 ± 17.3 vs. 63.5 ± 15.7, *P* <0.001).

### Population-based rates of ICU care

Approximately 0.72% of men and 0.47% of women were admitted to ICUs each year (Table 2). The overall predominance of men admitted to ICUs as indicated by the unadjusted male:female rate ratio of 1.56 (95% confidence interval (CI) 1.53 to 1.58) becomes even more marked after adjusting for age (rate ratio 1.75, 95% CI 1.64 to 1.88).

**Table 2:** Unadjusted and age-adjusted rates of ICU care per 1,000 population, by year and sex

***Year***	***Unadjusted***			***Age-adjusted***		
	***Male***	***Female***	***M:F ratio***	***Male***	***Female***	***M:F ratio***
1999	7.79	4.97	1.57	8.46	4.63	1.83
2000	8.06	5.30	1.52	8.75	4.90	1.79
2001	7.41	4.98	1.49	8.07	4.63	1.74
2002	7.04	4.51	1.56	7.60	4.21	1.81
2003	7.10	4.50	1.58	7.64	4.21	1.82
2004	6.95	4.49	1.55	7.53	4.20	1.79
2005	6.81	4.42	1.54	7.30	4.18	1.75
2006	6.88	4.41	1.56	7.36	4.14	1.78
2007	7.03	4.30	1.63	7.45	4.06	1.83
Unweighted average	7.23	4.65	1.56	7.80	4.35	1.75
Average yearly change in rates	−0.13*	−0.11*	0.008†	−0.16*	−0.09*	−0.0005†

*P value <0.01; †*P* >0.05 obtained from linear regression of yearly values.

While admission rates were lower when restricting consideration to the high-intensity ICUs, male:female rate ratios were higher. Unadjusted values were 0.61% for men and 0.37% for women (sex ratio 1.63, 95% CI 1.60 to 1.66), while age-adjusted rates were 0.64% for men and 0.35% for women (sex ratio 1.82, 95% CI 1.71 to 1.95).

**Figure 1:**
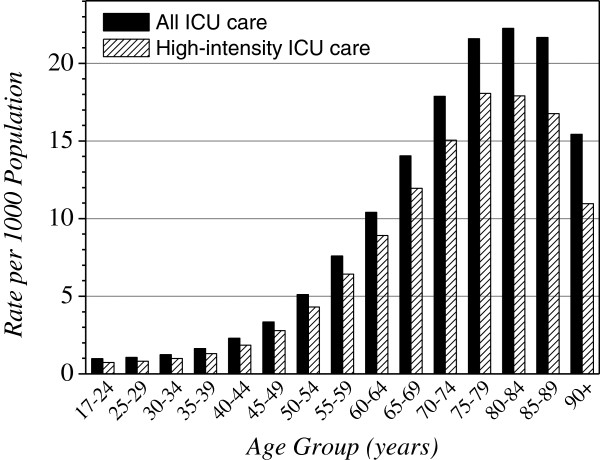
**Unweighted average, from 1999 to 2007, of age-specific rates of ICU care.**

Rates of ICU care differed substantially according to age; they rose rapidly after age 40, peaked at age 75 to 80, and then declined for the oldest age groups (Figure 1). Each year approximately 2% of Manitobans over age 70 were admitted to an ICU.

**Figure 2:**
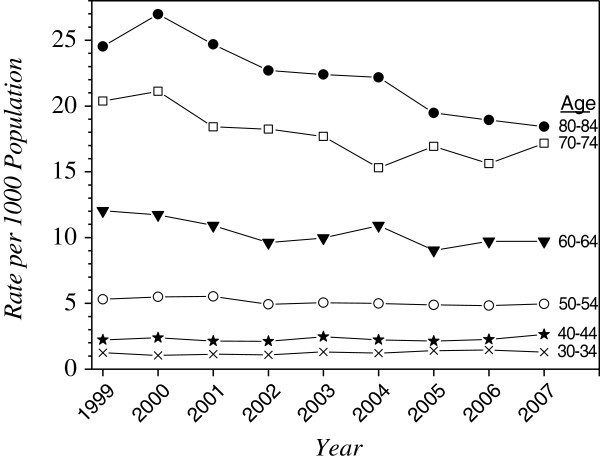
Selected age-specific rates of ICU care, by year.

We identified two temporal trends in the rates of ICU care. First, rates significantly declined over time for both sexes, though no trends were observed in the male: female rate ratios (Table 2). Second, these changes were age-dependent, with older age groups showing larger decreases over time (Figure 2, in Additional file 1: Table S2).

**Figure 3:**
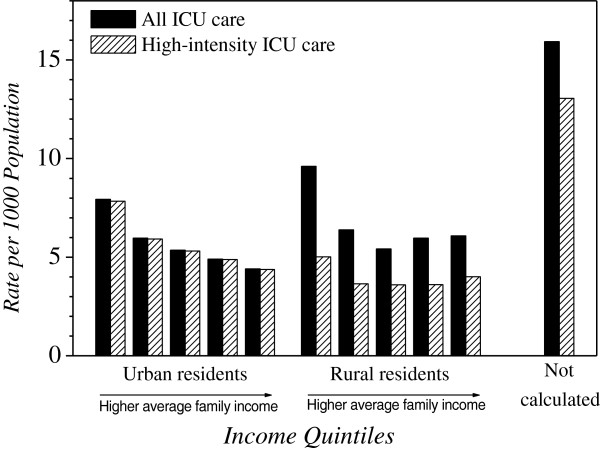
Unweighted average, from 1999 to 2007, of age-adjusted rates of ICU care, by socioeconomic status.

Analysis of rates of ICU care according to socioeconomic status (SES) showed significantly higher rates among those with lower average household income, in both urban and rural areas (Figure 3). The highest rates of ICU care were seen among residents of chronic care facilities and other institutions. In addition, while rural residents had higher rates of all ICU care, they had lower rates of admission to the high-intensity urban ICUs. For example, the age-adjusted rate per 1,000 population of admission to all ICUs among the poorest rural residents was higher than for the poorest urbanites (9.60 vs. 7.93, *P* <0.0001); in contrast, age-adjusted rates of admission to the high-intensity ICUs was lower for rural compared to urban residents (5.02 vs. 7.84, *P* <0.0001).

## Discussion

We performed a population-based analysis of ICU bed supply and utilization in an entire Canadian province, including trends over time. Findings include a high rate of ICU recidivism, slow but noticeable trends in ICU utilization, and differences in rates of ICU care by sex, age, and socioeconomic status.

The 9.8 adult ICU beds per 100,000 population in Manitoba in 2007 can be compared to 14.8 for Ontario [23], 13.5 for all of Canada (excluding Quebec and Manitoba), and 3.5 to 24.6 for other selected developed countries [1]. Although Manitoba ICU bed counts prior to 2007 are not available, total acute care beds per 100,000 population fell from 358 in 1999 to 325 in 2007. Few previous studies have reported population-based rates of ICU care. Manitoba’s rate is approximately double that reported in the Calgary health region of the Canadian province of Alberta [24]. Though the Calgary data excluded admissions to coronary care units, this can only explain a minority of the difference, as such patients comprised just 20% of admissions to Winnipeg ICUs. On the other hand, age-specific rates reported in Olmsted County, Minnesota are approximately double those in Manitoba [25]. There are several possible explanations for such large differences in rates of ICU care between jurisdictions. First, accuracy of identifying ICU use might differ; in our data we have validated the identification of ICU admission [20]. Second, definitions of what constitutes an ICU bed might differ [26]. Third, it could reflect true variation in the underlying rates of critical illness. Though this is suggested by the large variations in reported rates of severe sepsis [27-30], and the adult respiratory distress syndrome [31,32], divergent disease definitions and administrative data coding likely account for much of those differences. Also, it seems unlikely that Manitobans experience critical illness at a rate double or half that of people living within 1,300 km in Alberta or Minnesota. Last, jurisdictions may have very different thresholds for admitting patients to ICUs, using whatever ICU beds they possess [33]. This is supported by the finding that though the United States has substantially more ICU beds than Canada [1], it appears to use them to care for less severely ill patients, as indicated by 16 to 34% rates of mechanical ventilation [1,34,35], vs. over 50% in Canada [1,36].

The male predominance of ICU patients across we observed has been seen in most [24,37-39], but not all [25] prior studies. Although there has been concern that this phenomenon represents a disparity in access for women to ICU care, alternative explanations include higher rates of underlying critical illness among men, and lower willingness among women to receive the type of aggressive care provided in ICUs. As an example of the former, Fransoo *et al*. showed that higher rates of cardiac catheterization among men are largely explained by their higher rates of heart attack, [40] consistent with the known higher rate of cardiovascular disease known in men [41]. Indeed, in a preliminary analysis we found that men were much more likely than women to be admitted to an ICU in Manitoba with a cardiovascular primary diagnosis (58.8 vs. 48.0%, *P* <0.0001; in Additional file 1: Table S3).

We identified two age-related phenomena in the population-based rates of ICU care. First, cross-sectional rates increased steeply with age, peaking at approximately age 80, and then declined as age increased further. This pattern has been seen in another part of Canada [24], but not in a US locale, where the age-specific rates continued to rise in the highest age strata [25]. Second, longitudinal analysis over our nine-year study period showed that rates of ICU care declined among patients aged 50 and older, with faster declines among older age groups. While this could be due to declining rates of critical illness in middle age or beyond, it could alternatively reflect changes in older peoples’ willingness for ICU care, or lower ICU access afforded to older critically ill persons. Also, in an observation that has not been previously reported, we found that population-based rates of critical illness were systematically higher for people in lower income strata. Since this was true for both urban and rural dwellers, and Manitoba has universal health care insurance, it is not readily explained by differential access to ICU care. The only prior evaluation of ICU access related to SES that we could identify found no relationship among patients admitted to a single hospital in Vancouver [38]. However, several publications from countries with universal health insurance have shown that rates of hospitalization have the same income gradient as we observed for ICU admission [42,43].

The main limitation of this study is that because it was restricted to a single province in a single country it may not reflect the situation in other jurisdictions. Although the issue of generalizability is partly addressed by the above comparison of our findings to those of prior studies, unlike data on a treatment for a disease, the value of analyses of health services/health systems is not primarily about being generalizable. Instead, a main value and goal of studying the structural aspects of health care systems is to better understand the factors that determine their performance. Systems of ICU care, like all health systems, are so complex that making comparisons between them is a major component of working to understand the optimal way to structure them.

## Conclusions

In this population-based study of an entire Canadian province we found that approximately one-half of 1% of adults were admitted to ICUs each year. Men substantially outnumbered women in ICUs, with 0.72% of men and 0.47% of women admitted to ICUs yearly. ICU admission rates rose rapidly with age, but then declined among the oldest people. People with lower incomes had higher rates of ICU admission. Although admission rates declined for both sexes over the nine years of this study, LOS rose such that cumulative ICU bed-days grew by 1.3% per year. There was a high rate of ICU recidivism, as approximately one-sixth of people admitted had been in an ICU before.

These findings add to the existing literature on the epidemiology of critical illness. Those responsible for health system planning need such data to understand current ICU needs, and to ensure that they are able to meet projected future needs. Also, identifying subgroups with disproportionately high utilization is the starting point for further research to illuminate the reasons for such phenomena, and to devise strategies for reducing critical illness in high-risk groups.

A comprehensive understanding of the epidemiology of critical illness includes not only information on utilization rates and patient demographics as presented here, but also details about the type and severity of illness, and outcomes. Planned future analysis of the Manitoba Integrated Critical Care Database will address those issues.

## Key messages

● Admission to ICUs was common, with approximately 1 in 200 adults admitted each year.

● There were substantial differences in rates of ICU admission, by sex, age, and income level.

● Although rates of admission fell over the study period, the average ICU length of stay grew, such that cumulative ICU bed-days also rose.

## Abbreviations

CI: Confidence interval; ICU: Intensive care unit; LOS: Length of stay; MICCDB: Manitoba integrated critical care database; SES: Socioeconomic status.

## Competing interests

The authors declare that they have no competing interests.

## Authors’ contributions

AG participated in study design, drafting the article, analysis and interpretation of data, and revising the article for intellectual content. KO participated in study design, interpretation of data, and revising the article for intellectual content. CR participated in study design, interpretation of data, and revising the article for intellectual content. MY participated in analysis and interpretation of data, and revising the article for intellectual content. RF participated in study design, drafting the article, analysis and interpretation of data, and revising the article for intellectual content. All authors read and approved the final manuscript.

## Supplementary Material

Additional file 1: Table S1 Age of patients admitted to ICUs, by year. **Table S2**. Linear trend over time in age-specific rates of ICU care, from 1999 to 2007. **Table S3**. Sex-specific number and percentage of ICU episodes, by diagnostic category, from 1999 to 2007. Click here for file
