# Supplementary material for: Epidemiology of critically ill patients in intensive care units: a population-based observational study
Source: Crit Care. 2013 Sep 30;17(5):R212. doi: 10.1186/cc13026 (PMC4056438; doi:10.1186/cc13026)
Supplement: Additional file 1: Table S1 — Age of patients admitted to ICUs, by year. Table S2. Linear trend over time in age-specific rates of ICU care, from 1999 to 2007. Table S3. Sex-specific number and percentage of ICU episodes, by diagnostic category, from 1999 to 2007. [file cc13026-S1.doc]

**ADDITIONAL FILE**

Additional file Table S1. Age of patients admitted to ICUs, by fiscal year.

| *Fiscal year* | *# of unique persons*  *in that year* | *-----------------------Age-----------------------* | | | |
| --- | --- | --- | --- | --- | --- |
|  |  | *Mean* | *SD* | *Median* | *Interquartile range* |
| 1999 | 5,534 | 65.0 | 16.2 | 68.0 | 55.0- 77.0 |
| 2000 | 5,833 | 65.4 | 16.3 | 69.0 | 56.0 - 77.0 |
| 2001 | 5,437 | 65.4 | 15.9 | 68.0 | 55.0 - 77.0 |
| 2002 | 5,100 | 64.7 | 16.2 | 68.0 | 54.0 - 77.0 |
| 2003 | 5,168 | 64.4 | 16.4 | 67.0 | 54.0 - 77.0 |
| 2004 | 5,110 | 64.3 | 16.6 | 66.0 | 54.0 - 77.0 |
| 2005 | 5,074 | 63.8 | 16.5 | 66.0 | 53.0 - 77.0 |
| 2006 | 5,136 | 64.0 | 16.8 | 66.0 | 54.0 - 77.0 |
| 2007 | 5,224 | 63.7 | 16.5 | 66.0 | 54.0 - 76.0 |

**Additional file Table S2*.*** Linear trend over time in age-specific rates of ICU care, over 1999-2007.

|  | -----All ICU care----- | | --High-intensity ICU care-- | |
| --- | --- | --- | --- | --- |
| *Age range (years)* | *Coefficient* | *p-value* | *Coefficient* | *p-value* |
| 17-24 | 0.0017 | 0.90 | -0.00067 | 0.94 |
| 25-29 | -0.00017 | 0.99 | -0.0036 | 0.78 |
| 30-34 | 0.035 | 0.046 | 0.027 | 0.03 |
| 35-39 | 0.032 | 0.34 | 0.028 | 0.28 |
| 40-44 | 0.023 | 0.34 | 0.038 | 0.07 |
| 45-49 | -0.033 | 0.25 | -0.037 | 0.14 |
| 50-54 | -0.077 | 0.01 | -0.044 | 0.15 |
| 55-59 | -0.16 | 0.01 | -0.11 | 0.06 |
| 60-64 | -0.30 | 0.01 | -0.22 | 0.04 |
| 65-69 | -0.49 | <.001 | -0.41 | <.001 |
| 70-74 | -0.59 | 0.01 | -0.46 | 0.008 |
| 75-79 | -0.82 | 0.001 | -0.65 | 0.002 |
| 80-84 | -0.99 | <.001 | -0.82 | <.001 |
| 85-89 | -0.43 | 0.15 | -0.36 | 0.19 |
| ≥90 | -0.57 | 0.01 | -0.45 | 0.008 |

**Additional file Table S3*.*** Sex-specific number and percentage of ICU episodes, by diagnostic category, over 1999-2007.

| *Diagnostic category* | *Male* | *Female* | *Both sexes* |
| --- | --- | --- | --- |
| Cardiovascular diagnoses | 19,029 (58.8%) | 10,455 (48.0%) | 29,484 (54.5%) |
| Non-cardiovascular diagnoses | 13,321 (41.2%) | 11,335 (52.0%) | 24,656 (45.5%) |
| All diagnoses | 32,350 (100%) | 21,790 (100%) | 54,140 (100%) |
